# Associations between adherence to the Taiwan Daily Food Guide and psychiatric morbidity: A population-based study in Taiwan

**DOI:** 10.3389/fpsyt.2022.1022892

**Published:** 2022-11-01

**Authors:** Ming-Chieh Li

**Affiliations:** Department of Health Promotion and Health Education, College of Education, National Taiwan Normal University, Taipei, Taiwan

**Keywords:** mental health, psychiatric morbidity, psychiatric disorders, dietary guidelines, Daily Food Guide

## Abstract

**Background:**

Mental health has become a public health concern worldwide, and the number of affected individuals is rising. Therefore, further research must be conducted to identify potential risk factors to develop optimal prevention strategies to mitigate mental health disorders.

**Methods:**

Using Taiwanese Nutrition and Health Survey data collected from 2013–2016, we conducted a cross-sectional study to examine whether adherence to the Taiwan Daily Food Guide affects mental health conditions. Study participants were adults aged ≥19 years. The dietary assessment was conducted using a validated food frequency questionnaire. The presence of psychiatric morbidity was defined as a five-item Brief Symptom Rating Scale (BSRS-5) score of ≥10. Logistic regression models were used to determine whether Taiwan Daily Food Guide adherence was related to the presence of psychiatric morbidity.

**Results:**

After adjusting for potential confounders, we observed protective associations between adherence to the Taiwan Daily Food Guide and psychiatric morbidity risk.

**Conclusion:**

The Taiwan Daily Food Guide might reduce the risk associated with psychiatric morbidity and could be a reference for developing a national food guide for mental health.

## Introduction

Mental health has become an increasingly burdensome public health challenge worldwide (1). The number of disability-adjusted life-years (DALYs) owing to mental disorders increased from 80.8 million in 1990 to 125.3 million globally in 2019 (2), remaining within the top ten causes of burden. Therefore, identifying possible risk factors is essential for developing effective prevention strategies against mental health conditions.

Dietary change might be a potential strategy for preventing mental health conditions. It is particularly attractive because it is a modifiable factor that can be controlled at all times. Previous studies revealed that certain dietary patterns are associated with a reduced risk of mental health conditions. For example, a meta-analysis suggested that a dietary pattern characterized by more frequent consumption of fruits and vegetables, whole grains, fish, olive oil, low-fat dairy, and antioxidants; and less frequent intake of animal foods was associated with reduced risk of depression. In contrast, frequent consumption of red and/or processed meat, refined grains, sweets, high-fat dairy products, butter, potatoes, and high-fat gravy; and less frequent intake of fruits and vegetables are associated with an elevated risk of depression (3). Some studies on Asian populations have revealed dietary patterns that are likely beneficial for mental health. For example, a cross-sectional study conducted in China revealed that after adjusting for age, gender, maternal and paternal education, family income, body mass index (BMI), and physical activity, consuming large amounts of snacks and animal foods increases the risk of mental disorders (4). Another cross-sectional study conducted in Japan showed that after adjusting for age, sex, workplace, marital status, BMI, job position, occupational physical activity, current smoking, non-job physical activity, history of hypertension and diabetes mellitus, and total energy intake, a dietary pattern characterized by more frequent consumption of vegetables and fruits, mushrooms, and soy products reduces the risk of depressive symptoms (5). Similarly, a case-control study among Korean adolescent girls indicated that after adjusting for menstrual regularity and energy intake, depression was significantly negatively associated with green vegetable and fruit intake. In contrast, depression was positively associated with the consumption of instant and processed foods (6). In summary, dietary patterns characterized by plant-based foods may benefit mental health.

Although certain dietary patterns might reduce the risk of mental conditions, governments and policymakers rarely state that a specific dietary pattern should be adopted. A general dietary guideline or daily food guide is usually adopted, such as those constructed by the US Department of Health and Human Services (DHHS), the US Department of Agriculture (USDA), and the World Health Organization (7, 8). This remains true in Taiwan. Although some Taiwanese studies have identified specific dietary patterns that affect health (9–11), it remains unclear whether Taiwan's daily food guide prevents disease. Further, the inclusion of particular recommendations may prevent some diseases but not others (12). In fact, some have argued that a dietary guideline may be more harmful than beneficial (13, 14). Therefore, instead of identifying a dietary pattern related to mental health, we aimed to examine whether adherence to the Taiwan Daily Food Guide reduced the risk of mental health conditions.

## Materials and methods

The Nutrition and Health Survey in Taiwan (NAHSIT) 2013–2016 is a nationwide representative survey aimed at assessing the nutritional status of the general Taiwanese population. Survey methods employed have been described elsewhere (9, 15, 16). Briefly, study participants were selected *via* the application of three-stage probability sampling covering 359 townships or city districts. Throughout the first stage, the probability proportional to size sampling method was utilized to select eight primary sampling units (townships and city districts) and 160 townships or city districts. In the second stage, households were randomly selected to construct sampling clusters within each selected primary sampling unit. Finally, door-to-door visits were conducted by trained interviewers until the required number of sex and age groups were reached. Because seasonal variations may affect dietary consumption, the NAHSIT used a Latin square design to ensure that data collection times were spaced evenly throughout all four seasons. All participants were invited to a temporary health examination station and underwent a physical examination.

Study participants of NAHSIT 2013–2016 were contacted by door-to-door visits. In the NAHSIT survey, 11,072 participants aged 2 months and above were included. The response rate for the household visit was 77.2% in 2016. The present study restricted participants to adults aged ≥19 years (*n* = 5,770, 52.1%), a designation in accordance with the definition of an adult in the newest version of the Taiwan Daily Food Guide. Among them, 2,534 participants (43.9%) who had undergone physical examination and had complete demographic and dietary data were included in the final analysis. Demographic and lifestyle data, including age, sex, education, marital status, smoking status, alcohol intake, exercise, and self-reported medical history, were obtained *via* face-to-face interviews. The study protocol was approved by the China Medical University and Hospital Research Ethics Center (CRREC-108-136). The need for informed consent was confirmed by the research ethics center.

### Psychiatric morbidity

A self-administered questionnaire (five-item Brief Symptom Rating Scale, BSRS-5) was used to determine the prevalence of psychiatric morbidity. The BSRS-5 is a five-item Likert scale that assesses the following: 1) difficulty falling asleep (insomnia), 2) feeling low in mood (depression), 3) feeling tense or keyed up (anxiety), 4) feeling easily annoyed or irritated (hostility), and 5) feeling inferior to others (inferiority) (17). Scores given for each item ranged from 0 to 4. According to a guideline in Taiwan, BSRS-5 scores were categorized as “mild” psychiatric morbidity with a score of 6–9, “moderate” with a score of 10–14, and “severe” with a score more than 15 (17, 18). The optimal cut-off values were suggested in a validation study (19). Studies have indicated that the BSRS-5 is a high-quality assessment tool that may be used to detect psychiatric morbidity and suicidal ideation in both community and medical settings (20, 21). In the current study, the presence of psychiatric morbidity was defined as a BSRS-5 score ≥ 10, which indicates moderate to severe psychiatric morbidity that may require psychiatric counseling (22, 23).

### Measurement of adherence to the Daily Food Guide

The Department of Health in Taiwan (now the Ministry of Health and Welfare in Taiwan) established the first edition of the Daily Food Guide in 1984 (24). The Ministry of Health and Welfare in Taiwan released the most recently updated edition of the Daily Food Guide in 2018, which included changes aimed at preventing nutrient deficiencies. The Taiwanese Daily Food Guide was constructed not only based on epidemiologic evidence but also to reduce the risk of chronic diseases. Guidelines recommend minimal servings for the following six food groups based on individual daily energy needs (15): 1) cereals and whole grains; 2) protein-rich foods (soybean, fish, egg, and meat); 3) dairy products; 4) vegetables; 5) fruits, and 6) fats, oils, and nuts. Face-to-face dietary interviews were performed by trained interviewers using a 79-item food-frequency questionnaire (FFQ). The FFQ was then divided into 23 food groups based on nutritional content and characteristics (25, 26). A similar simplified FFQ was validated by comparing it with data obtained by 24-h dietary recalls and nutritional biomarkers (10, 27) and showed good correlation coefficients.

Participant adherence levels to the Daily Food Guide were determined by a previously constructed Daily Food Guide index (15). Supplementary Table S1 shows the Taiwan Daily Food Guide (28), which was translated by the author. We calculated a single score for each study participant, which allowed us to rank their adherence levels to the Daily Food Guide (28). Because fat and oil intakes were not adequately assessed using the FFQ, we did not assess levels of adherence to fat and oil intake among study participants. The appropriate quantity of each food group that the participants should consume was determined by their estimated energy needs. Energy needs were estimated based on level of physical activity, resting metabolic rate, and healthy weight (28, 29). The score that a person received in any food category was determined by their consumption of an appropriate number of servings based on their daily energy needs (Supplementary Table S1). For example, a person who consumed the recommended number of servings from any food group received a score of 1 for that particular food group category. In contrast, a person who consumed no servings of a specific food group received a score of 0. Each score was calculated based on the proportion of recommended servings consumed (15).

### Statistical analysis

Only participants with complete data were included in the final analysis. The participants were divided into five groups according to their Daily Food Guide index scores. If participants received a total score >6, they were placed in group 5, indicating that they had consumed more than the total number of recommended servings. The remaining participants who received scores ≤ 6 were then divided into four groups by the quartile of the Daily Food Guide index scores. The association between demographic characteristics and the prevalence of psychiatric morbidity was evaluated using chi-square or Fisher's exact tests (categorical variables). Potential confounding factors were selected based on prior knowledge and their relationship with exposures (adherence levels) and outcomes (the presence of psychiatric morbidity) (30). All the models were adjusted for age, sex, BMI, education level, alcohol intake, smoking status, marital status, family income, and physical activity.

A logistic regression model was applied to examine whether the Daily Food Guide index score was associated with the presence of psychiatric morbidity (present vs. not present). A *p*-value < 0.05 was considered statistically significant. All analyses were conducted using SAS software (version 9.4; SAS Institute, Cary, NC, USA).

## Results

A total of 2,534 participants with no missing data were included in the final analysis. Among them, 402 (15.9%) reported psychiatric morbidities. A comparison of the demographic characteristics of the two groups is listed in Table 1. Compared with those without psychiatric morbidity, participants who reported psychiatric morbidity were more likely to be women than men (60.95 vs. 49.62%, respectively), younger vs. older than 30 (22.39 vs. 12.34%, respectively), have a BMI value of < 24 than ≥24 (56.47 vs. 50.00%, respectively), be married or live together than single (22.89 vs. 15.43%, respectively), and have median to high physical activity than low activity levels (67.41 vs. 59.89%, respectively).

**Table 1:** Demographic characteristics of study participants (*n* = 2,534).

**Variables**	**Participants without psychiatric morbidity**	**Participants with psychiatric morbidity**	***p*-value**
	***n* = 2,132**	***n* = 402**	
**Sex**			
Men	1074 (50.38)	157 (39.05)	< 0.0001
Women	1058 (49.62)	245 (60.95)	
**Age**			
Age ≤ 30	263 (12.34)	90 (22.39)	< 0.0001
40 ≥ age > 30	215 (10.08)	64 (15.92)	
50 ≥ age > 40	280 (13.13)	71 (17.66)	
65 ≥ age > 50	714 (33.49)	96 (23.88)	
Age > 65	660 (30.96)	81 (20.15)	
**Body mass index (BMI)**			
BMI < 24	1066 (50.00)	227 (56.47)	0.04
27 > BMI ≥ 24	558 (26.17)	84 (20.90)	
BMI ≥ 27	508 (23.83)	91 (22.64)	
**Education**			
Elementary school	1072 (50.28)	199 (49.5)	0.06
Junior high and high school	616 (28.89)	100 (24.88)	
College or above	444 (20.83)	103 (25.62)	
**Drink**			
No	1002 (47)	185 (46.02)	0.83
Rarely	856 (40.15)	161 (40.05)	
Frequently	274 (12.85)	56 (13.93)	
**Smoke**			
No	1,500 (70.36)	293 (72.89)	0.59
Ever smoke	313 (14.68)	54 (13.43)	
Current smoke	319 (14.96)	55 (13.68)	
**Marital status**			
Married or lived together	329 (15.43)	92 (22.89)	< 0.001
Single	1486 (69.7)	250 (62.19)	
Divorced, separated, widowed, or refused to answer	317 (14.87)	60 (14.93)	
**Family income**			
Income < NT $10,000	173 (8.11)	37 (9.2)	0.26
NT $40,000>Income ≥ NT $10,000	449 (21.06)	85 (21.14)	
NT $80,000>Income ≥ NT $40,000	519 (24.34)	109 (27.11)	
Income ≥ NT $80,000	488 (22.89)	96 (23.88)	
Do not know or refuse to answer	503 (23.59)	75 (18.66)	
**Physical activity**			
Low	855 (40.1)	131 (32.59)	0.02
Median	1224 (57.41)	258 (64.18)	
High	53 (2.48)	13 (3.23)	

NT: The New Taiwan dollars.

Table 2 shows the relationship between the Daily Food Guide adherence score and the risk of psychiatric morbidity. After adjusting for age, sex, BMI, education level, alcohol intake, smoking status, marital status, family income, and physical activity, negative associations were found between Daily Food Guides adherence and psychiatric morbidity. The odds ratio (OR) for participants who were in the highest quartile for recommended total serving consumption was 0.60 [95% confidence interval (CI): 0.41–0.87] compared with those in the lowest quartile. Participants consuming a higher number of total servings than recommended were at reduced risk of psychiatric morbidity (OR: 0.44, 95% CI: 0.30–0.63). In addition, men (OR: 0.52, 95% CI: 0.39–0.69) aged 50–65 years (OR: 0.38, 95% CI: 0.24–0.61) or more than 65 years (OR: 0.26, 95% CI: 0.15–0.47), and those who did not know or refused to report their family income (OR: 0.54, 95% CI: 0.34–0.85) were at reduced risk of psychiatric morbidity.

**Table 2:** The relationship between the Daily Food Guide adherence score and the risk of psychiatric morbidity.

**Variables**	***n* = 2,534**
**Diet score**	
**Total servings equal to or lower than recommendation**
Group 1	**Reference**
Group 2	1.01 (0.73–1.41)
Group 3	0.77 (0.54–1.09)
Group 4	0.60 (0.41–0.87)
**Total servings higher than recommendation**
Group 5	0.44 (0.30–0.63)
**Sex**
Women	**Reference**
Men	0.52 (0.39–0.69)
**Age**
Age ≤ 30	**Reference**
40 ≥ age > 30	0.77 (0.50–1.19)
50 ≥ age > 40	0.69 (0.44–1.09)
65 ≥ age > 50	0.38 (0.24–0.61)
Age > 65	0.26 (0.15–0.47)
**Body mass index (BMI)**
BMI < 24	**Reference**
27 > BMI ≥ 24	0.89 (0.67–1.18)
BMI ≥ 27	0.96 (0.72–1.26)
**Education**
College or above	**Reference**
Junior high and high school	1.08 (0.69–1.68)
Elementary school	0.92 (0.68–1.25)
**Drink**
Non	**Reference**
Rarely	0.99 (0.77–1.28)
Frequently	1.29 (0.89–1.87)
**Smoke**
Non	**Reference**
Ever smoke	1.36 (0.93–1.99)
Current smoke	0.90 (0.62–1.30)
**Physical activity**
Low	**Reference**
Median	0.82 (0.60–1.13)
High	0.86 (0.43–1.71)
**Marital status**
Married or lived together	**Reference**
Single	0.87 (0.59–1.29)
Divorced, separated, widowed, or refused to answer	1.15 (0.82–1.61)
**Family income**
Income < NT $10,000	**Reference**
NT $40,000 > income ≥ NT $10,000	0.78 (0.50–1.22)
NT $80,000 > income ≥ NT $40,000	0.74 (0.46–1.17)
Income ≥ NT $80,000	0.71 (0.44–1.16)
Do not know or refuse to answer	0.54 (0.34–0.85)

NT: The New Taiwan dollars; All logistic models were adjusted for age, sex, BMI, education level, alcohol drinking, smoking status, physical activity, marital status, and family income.

Relationships between adherence to individual food group index recommendations and the presence of psychiatric morbidity are shown in Table 3. After adjusting for potential confounding factors, a negative association was found between adherence to food group 4 index recommendation (vegetables) and risk of psychiatric morbidity. ORs for participants with score between 0.5–1 and >1 were 0.69 (95% CI: 0.51–0.95) and 0.64 (95% CI: 0.46–0.88), respectively.

**Table 3:** Relationships between adherence to individual food group index recommendations and the presence of psychiatric morbidity.

**Variable**	***n* = 2,534**
**Food group 1 (cereals and whole grains)**
Score < 0.5	**Reference**
0.5 ≤ score ≤ 1	0.56 (0.31–1.02)
Score > 1	0.86 (0.38–1.95)
**Food group 2 (protein-rich foods)**
Score < 0.5	**Reference**
0.5 ≤ score ≤ 1	0.80 (0.63–1.02)
Score > 1	1.22 (0.77–1.94)
**Food group 3 (dairy products)**
Score < 0.5	**Reference**
0.5 ≤ score ≤ 1	0.83 (0.62–1.10)
Score > 1	1.30 (0.69–2.44)
**Food group 4 (vegetables)**
Score < 0.5	**Reference**
0.5 ≤ score ≤ 1	0.69 (0.51–0.95)
Score > 1	0.64 (0.46–0.88)
**Food group 5 (fruits)**
Score < 0.5	**Reference**
0.5 ≤ score ≤ 1	0.79 (0.50–1.24)
Score > 1	0.80 (0.56–1.15)
**Food group 6 (nuts)**
Score < 0.5	**Reference**
0.5 ≤ score ≤ 1	1.15 (0.71–1.84)
Score > 1	0.74 (0.49–1.11)

Score = 1 means that the participants consume recommended servings of the food group; score = 0.5 means that the participants consume half of recommended servings of the food group. All logistic models were adjusted for age, sex, BMI, education level, alcohol drinking, smoking status, physical activity, marital status, and family income.

We tested whether the association between adherence levels and the risk of psychiatric morbidity was modified by age, BMI, and smoking status by introducing cross-product terms to the models. When there was a suggestive interaction effect (*p-*value < 0.10), stratified analysis was further conducted. However, we found no evidence that age, smoking, or BMI modified the relation between adherence levels and the risk of psychiatric morbidity.

## Discussion

In this study, we observed that participants who reported good adherence to the Daily Food Guide were at reduced risk of psychiatric morbidity. A protective effect remained in participants who consumed more than recommended. This protective effect may be mainly due to increased levels of vegetable consumption. Consumption of cereals, whole grains, and fruits was also associated with reduced psychiatric morbidity risk; however, the findings were not statistically significant. In addition, men and older participants are likely at reduced risk of psychiatric morbidity. Although it is not clear why some participants have lower adherence levels, a study in Taiwan has suggested that people who were males, younger, less educated, divorced, separated or widowed, and had lower family income was associated with a lower adherence level to the Daily Food Guide (15). We suggest that health educators could develop educational programs focusing on different demographic groups to improve adherence levels. Future qualitative studies were recommended to better understand reasons for non-adherent behavior.

The findings of the present study are in line with those of previous studies that revealed that vegetable intake reduces the risk of mental health conditions, including depression and anxiety (3, 4, 31). Several studies have also suggested that the adoption of a Dietary Approach to Stop Hypertension (DASH) diet, which is characterized by high vegetable, fruit, and low-fat dairy product, and low saturated fat, total fat, and cholesterol recommendations, is protective of mental health (32–36). Observed associations are likely explained by the following: 1) increased consumption of vegetables that contain high levels of antioxidants might protect against depression. Previous studies have indicated that a reduced level of oxidative stress reduces the risk of neuronal damage in the hippocampus, consequently reducing the risk of developing depression (37, 38); or 2) mental health status might affect food preference. We could not determine which explanation is accurate owing to its cross-sectional study design. We also found a protective effect between adherence to food group 5 index recommendation (fruits) and risk of psychiatric morbidity. However, the effect was not significant. The reason underlying this finding might be that most fruits contain sugar, and a meta-analysis has shown that less healthy dietary patterns, including higher consumption of the sugar-sweetened beverage, were associated with severe mental illness (39).

This study has several strengths and limitations. A fair sample size allowed us to assess possible associations between adherence to the Daily Food Guide and the risk of psychiatric morbidity. In addition, the population-based design allowed us to generalize the findings to the general population of Taiwan. Psychiatric morbidity was assessed using a valid and effective screening tool. A limitation of this study included its cross-sectional design; therefore, causality could not be established. The associations warrant further prospective investigation to establish causality. Further, the use of a one-time dietary or psychiatric assessment may have introduced non-differential misclassification of exposures and outcomes. Further, the use of a one-time dietary or psychiatric assessment may have introduced non-differential misclassification of exposures and outcomes. For example, some participants might have serious psychiatric symptoms but were not willing to report them. As a result, they were classified into the control group and resulting in a misclassification of outcomes. However, non-differential misclassification usually biases toward the null hypothesis, tending to minimize the associations, which suggests that true effects might have been underestimated in this study.

## Conclusions

In summary, adherence to the Taiwan Daily Food Guide may help reduce the risk of psychiatric conditions. The Taiwan Daily Food Guide might reduce the risk associated with psychiatric morbidity and can be a reference for developing a national food guide for mental health. Further prospective cohort studies will be needed to verify these findings.

## Data availability statement

The original contributions presented in the study are included in the article/Supplementary materials, further inquiries can be directed to the corresponding author.

## Ethics statement

This study was approved by the China Medical University and Hospital Research Ethics Center (CRREC-108-136). The patients/participants provided their written informed consent to participate in this study.

## Author contributions

M-CL contributed to the conception and design of the study, performed the statistical analysis and organized the database, wrote the first draft of the manuscript, and contributed to manuscript revision, read, and approved the submitted version.

## Funding

M-CL was supported by the China Medical University, Taiwan (CMU108-N-12) and the National Science and Technology Council, Taiwan (NSTC 111-2410-H-003-100-SSS).

## Conflict of interest

The author declares that the research was conducted in the absence of any commercial or financial relationships that could be construed as a potential conflict of interest.

## Publisher's note

All claims expressed in this article are solely those of the authors and do not necessarily represent those of their affiliated organizations, or those of the publisher, the editors and the reviewers. Any product that may be evaluated in this article, or claim that may be made by its manufacturer, is not guaranteed or endorsed by the publisher.
