# Supplementary material for: Associations between adherence to the Taiwan Daily Food Guide and psychiatric morbidity: A population-based study in Taiwan
Source: Front Psychiatry. 2022 Nov 1;13:1022892. doi: 10.3389/fpsyt.2022.1022892 (PMC9664218; doi:10.3389/fpsyt.2022.1022892)
Supplement: Supplementary file 1 [file Table_1.DOCX]

**Table S1.** The Daily Food Guide for adults in Taiwan.

|  | **Daily energy needs (kcal/day)** | | | | | |  |
| --- | --- | --- | --- | --- | --- | --- | --- |
| **Index item** | **≥ 1200** | **≥ 1500** | **> 1800** | **≥ 2000** | **≥ 2200** | **≥ 2500** | **> 2700** |
| Minimal servings from cereals and whole grains | 1.5 | 2.5 | 3 | 3 | 3.5 | 4 | 4 |
| Minimal servings from protein-rich foods | 3 | 4 | 5 | 6 | 6 | 7 | 8 |
| Minimal servings from dairy products | 1.5 | 1.5 | 1.5 | 1.5 | 1.5 | 1.5 | 2 |
| Minimal servings from vegetables | 3 | 3 | 3 | 4 | 4 | 5 | 5 |
| Minimal servings from fruits | 2 | 2 | 2 | 3 | 3.5 | 4 | 4 |
| Minimal servings from fats, oils, and nuts |  |  |  |  |  |  |  |
| Fats and oils | 3 | 3 | 4 | 5 | 5 | 6 | 7 |
| Nuts | 1 | 1 | 1 | 1 | 1 | 1 | 1 |
